# N,S‐Doped Porous Carbon Nanobelts Embedded with MoS_2_ Nanosheets as a Self‐Standing Host for Dendrite‐Free Li Metal Anodes

**DOI:** 10.1002/advs.202204232

**Published:** 2022-09-25

**Authors:** Binke Li, Weishan Cao, Shuaize Wang, Zhenjiang Cao, Yongzheng Shi, Jin Niu, Feng Wang

**Affiliations:** ^1^ State Key Laboratory of Chemical Resource Engineering Laboratory of Electrochemical Process and Technology for Materials Beijing University of Chemical Technology Beijing 100029 P. R. China; ^2^ Beijing Advanced Innovation Center for Soft Matter Science and Engineering Beijing University of Chemical Technology Beijing 100029 P. R. China

**Keywords:** carbon nanobelts, heteroatom doping, Li metal anode, lithiophilic site, self‐standing host

## Abstract

Metallic Li is one of the most promising anodes for high‐energy secondary batteries. However, the enormous volume changes and severe dendrite formation during the Li plating/stripping process hinder the practical application of Li metal anodes (LMAs). We have developed a sulfate‐assisted strategy to synthesize a self‐standing host composed of N,S‐doped porous carbon nanobelts embedded with MoS_2_ nanosheets (MoS_2_@NSPCB) for use in LMAs. In situ measurements and theoretical calculations reveal that the uniformly distributed MoS_2_ derivatives within the carbon nanobelts serve as stable lithiophilic sites which effectively homogenize Li nucleation and suppress dendrite formation. In addition, the hierarchical porosity and 3D nanobelt networks ensure fast Li‐ion diffusion and accommodate the volume change of Li deposits during the plating/stripping process. As a result, a Li–Li symmetric cell using the MoS_2_@NSPCB host operates steadily over 1500 h with an ultralow voltage hysteresis (≈24.2 mV) at 3 mA cm^−2^/3 mAh cm^−2^. When paired with a LiFePO_4_ cathode, the current collector‐free LMA endows the full cell with a high energy density of 460 Wh kg^−1^ and good cycling performance (with a capacity retention of ≈70% even after 1600 cycles at 10 C).

## Graphical abstract

N,S‐doped porous carbon nanobelts embedded with MoS_2_ nanosheets were prepared by a simple sulfate‐assistant method, showing superior performance as a self‐standing host for dendrite‐free lithium metal anodes in half cells and full cells. The modification mechanism of the host was detailedly investigated and revealed by in situ measurements combined with theoretical calculations.

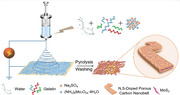

## 1 Introduction

The energy densities of Li‐ion batteries based on graphite anodes cannot meet the ever‐increasing energy demands of electric vehicles and power stations.^[^ ^1^, ^2^ ^]^ Metallic Li is considered an ideal anode material for high‐energy rechargeable batteries because of its ultrahigh theoretical specific capacity (3860 mAh g^−1^) and ultralow electrochemical potential (−3.04 V vs standard hydrogen electode).^[^ ^3^ ^]^ Unfortunately, the practical application of Li metal anodes (LMAs) involves two major challenges—undesirable dendrite formation and enormous volume change—which continuously decompose the electrolyte by destroying/rebuilding the solid electrolyte interface (SEI), and can even cause a short circuit leading to potential safety problems.^[^ ^4^, ^5^, ^6^, ^7^ ^]^

Many strategies have been proposed to improve LMAs, including designing liquid/solid electrolytes, constructing artificial SEIs, and introducing 3D hosts.^[^ ^8^, ^9^, ^10^, ^11^ ^]^ Prestoring Li in 3D hosts can efficiently regulate the electric fields governing Li‐ion transport resulting in uniform current distributions for Li nucleation and growth.^[^ ^12^, ^13^, ^14^, ^15^ ^]^ Carbon‐based frameworks are ideal hosts for high‐performance LMAs because of their excellent electrical conductivity and high electrochemical stability.^[^ ^16^ ^]^ However, the lithiophobic nature of carbon materials results in high barriers for Li nucleation, resulting in an inability to regulate subsequent Li growth.^[^ ^17^ ^]^ Although a variety of metal and non‐metal atoms and their compounds have been introduced into a carbon matrix in an attempt to change the surface chemistry of the carbon‐based hosts,^[^ ^18^, ^19^, ^20^ ^]^ some issues remain unresolved: i) complicated preparation procedures are usually required to ensure the efficient introduction of lithiophilic sites; ii) how to balance the conflicting requirements of high structural stability and high surface activity of lithiophilic sites; iii) hosts having both abundant lithiophilic sites and good mechanical properties are difficult to fabricate; iv) mechanisms of host modification in LMAs are not well understood.

We report the use of a simple sulfate‐assisted method to fabricate a new self‐standing host consisting of N,S‐doped carbon nanobelts with hierarchical porosity impregnated with MoS_2_ nanosheets (denoted as MoS_2_@NSPCB). The MoS_2_@NSPCB host has abundant metal/non‐metal lithiophilic sites facilitating uniform Li nucleation. Additionally, the hierarchical porosity and N,S‐doped carbon support endow the lithiophilic sites with good structural stability as well as high surface chemical activity. Moreover, the 3D conductive nanobelt networks ensure fast Li‐ion diffusion, preventing the formation of dendrites in the LMA during the plating/stripping process. LMAs based on the MoS_2_@NSPCB host show excellent performance in half cells. Furthermore, the current collector‐free LMAs endow full cells with high energy density, long cycling and fast‐charge/discharge performance.

## 2 Results and Discussion

**Figure 1:**
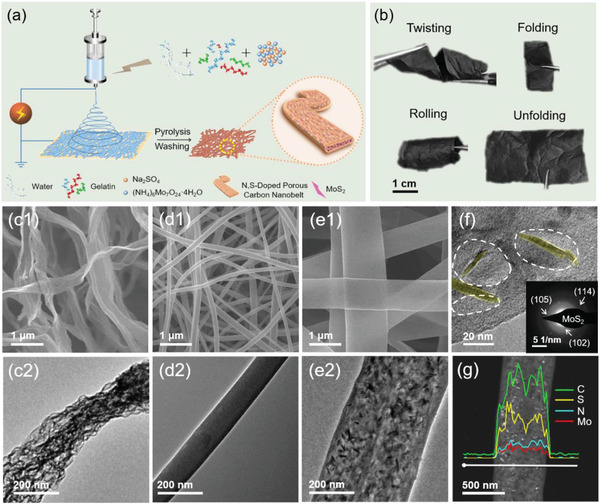
a) Schematic illustration of the synthesis of MoS_2_@NSPCB films. b) Digital photos showing the flexibility of the MoS_2_@NSPCB film in various states, including twisting, rolling, and folding. SEM images of (c1) NSPCB, (d1) MoO_x_@NCF, and (e1) MoS_2_@NSPCB. TEM images of (c2) NSPCB, (d2) MoO_x_@NCF, and (e2) MoS_2_@NSPCB. f) High‐resolution TEM image (inset: selected area electron diffraction pattern) and g) EDX linear scanning analysis of MoS_2_@NSPCB.

**Figure** **1**a shows a schematic illustration of the synthesis for the MoS_2_@NSPCB host. An aqueous solution of gelatin containing Na_2_SO_4_ and ammonium molybdate ((NH_4_)_6_Mo_7_O_24_·4H_2_O) was used to electrospin films, which were then pyrolyzed and washed with deionized water. In contrast to previously reported gelatin‐derived carbon films, the MoS_2_@NSPCB film shows superb flexibility as a self‐standing host (Figure 1b). Two other gelatin‐derived carbon samples were prepared using the same procedure, one without Na_2_SO_4_ and the other without ammonium molybdate. As shown in Figure S1 (Supporting Information), the former (denoted as MoO*_x_* @NCF) is also a self‐standing film, while the latter (denoted as NSPCB) is easily broken into small pieces, indicating that the introduction of ammonium molybdate improves the mechanical strength of the final samples due to the coordination between Mo centers and functional groups in gelatin.^[^ ^21^, ^22^ ^]^

X‐ray diffraction (XRD) and Raman spectroscopy confirm the presence of gelatin‐derived carbons in all three samples (Figure S2, Supporting Information). Characteristic peaks of MoS_2_ are observed in the XRD pattern and Raman spectrum of MoS_2_@NSPCB, while no characteristic peaks of Mo compounds are shown in those of MoO*_x_* @NCF. Due to the similar weight content of noncarbon materials for MoO*_x_* @NCF and MoS_2_@NSPCB (≈40%, Figure S3, Supporting Information), the metallic compound within MoO_x_@NCF is considered to have an amorphous structure. X‐ray photoelectron spectroscopy (XPS, Figure S4, Supporting Information) confirms that MoO*_x_* and MoS_2_ are present in MoO*_x_* @NCF and MoS_2_@NSPCB, respectively.^[^ ^23^ ^]^ This suggests that Na_2_SO_4_ acts as a sulfurizing reagent and reacts with molybdate ions to form MoS_2_. N species are observed in the XPS spectra of all the samples and additional S species are observed in the XPS spectra of NSPCB and MoS_2_@NSPCB, suggesting that Na_2_SO_4_ also plays a role as a dopant by introducing S heteroatoms into the carbon framework.

The addition of Na_2_SO_4_ not only affects the chemical composition but also regulates the structural morphology of the samples. Scanning electron microscopy (SEM) results (Figure 1c1–e1) show that NSPCB and MoS_2_@NSPCB have ultrathin nanobelt structures, while MoO*_x_* @NCF has a nanofiber structure. This suggests that Na_2_SO_4_ can influence the rheological or other properties of the solution and favor the formation of a nanobelt structure during the electrospinning process (Figure S5, Supporting Information). Transmission electron microscopy (TEM) images show that obvious pores exist in NSPCB and MoS_2_@NSPCB, while no pores can be observed in MoO_x_@NCF (Figure 1c2–e2). N_2_ adsorption/desorption measurements verify the hierarchical porosities of NSPCB and MoS_2_@NSPCB and the non‐porous structure of MoO*_x_* @NCF, suggesting that Na_2_SO_4_ also acts as a template for nanopore formation (Figure S6, Supporting Information). Well‐dispersed nanosheets are observed in MoS_2_@NSPCB (Figure 1f; and Figure S7, Supporting Information), which are confirmed to be MoS_2_ by selected area electron diffraction (Figure 1f, inset).^[^ ^24^, ^25^ ^]^ It should be noted that these MoS_2_ nanosheets are embedded within the porous carbon nanobelts since they are unobservable in the SEM image. Energy dispersive X‐ray (EDX) results of MoS_2_@NSPCB are displayed in Figure 1g; and Figure S7d (Supporting Information). The linear scanning curves exhibit a mesa‐like shape, characteristic of a nanobelt structure. The high dispersion of C, N, and S in the elemental mapping images indicate that N/S atoms are doped in the carbon nanobelts, consistent with the XPS results.

**Figure 2:**
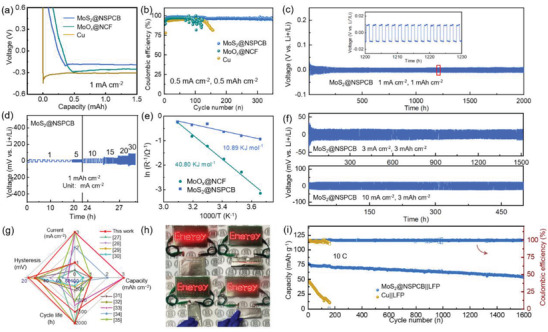
a) The voltage−capacity curves in the initial cycle and b) Coulombic efficiency (the initial cycle is not shown) of Li|Cu, Li|MoO*_x_* @NCF, and Li|MoS_2_@NSPCB half cells. c,d) Galvanostatic cycling performance of Li|Li symmetric cells using MoS_2_@NSPCB hosts at different current densities. e) Activation energy for Li‐ion diffusion with the MoS_2_@NSPCB and MoO*_x_* @NCF hosts. f) Galvanostatic cycling performance of Li|Li symmetric cells using MoS_2_@NSPCB hosts with plating/stripping capacity of 3 mAh cm^−2^. g) Comparison of the electrochemical performance of Li|Li symmetric cells using the MoS_2_@NSPCB host with the cells using other hosts. h) Digital photos of a MoS_2_@NSPCB‐Li|LiFePO_4_ pouch cell powering LED lights in different states. i) Cycling performance of the MoS_2_@NSPCB‐Li|LiFePO_4_ and Cu‐Li|LiFePO_4_ full cells.

The self‐standing films of MoS_2_@NSPCB and MoO_x_@NCF were used as hosts for LMAs. Li|Cu, Li|MoO*_x_* @NCF, and Li|MoS_2_@NSPCB half‐cells were assembled in order to compare the Li plating/stripping behaviors. As shown in **Figure** **2**a, the MoS_2_@NSPCB host displays the smallest overpotential for Li nucleation and plating in the initial cycle, corresponding to the lowest nucleation barrier and fastest Li‐ion migration.^[^ ^15^ ^]^ Moreover, the Li|MoS_2_@NSPCB cell performs the highest Coulombic efficiency of all the cells with a good cycling performance over 350 cycles at 0.5 mA cm^−2^ for 0.5 mAh cm^−2^ (Figure 2b). An average Coulombic efficiency of ≈99% was obtained at 1 mA cm^−2^ for 1 mAh cm^−2^ (Figure S8, Supporting Information), indicative of the high reversibility of Li plating/stripping in the MoS_2_@NSPCB host. Symmetric cells were assembled to evaluate the galvanostatic cyclic performance and the interfacial stability of the LMAs with and without the hosts. At a current of 1 mA cm^−2^ for 1 mAh cm^−2^, a Li|Li symmetric cell using the MoS_2_@NSPCB host shows a stable cycling life over 2000 h with an ultralow voltage hysteresis of ≈22.7 mV (Figure 2c). In contrast, Li|Li symmetric cells with the MoO_x_@NCF host and Cu foil display high voltage hysteresis of ≈73.0 and ≈53.0 mV, respectively (Figure S9, Supporting Information). A short‐circuit eventually occurred after ≈65 h, causing a sudden drop in the voltage hysteresis.

The MoS_2_@NSPCB host also endows the Li|Li cell with an excellent rate performance (Figure 2d), with stable working even at 30 mA cm^−2^ with a low voltage overpotential below 100 mV. Electrochemical impedance spectroscopy (EIS) was employed to investigate the kinetics of charge transfer and Li‐ion diffusion (Figure S10, Supporting Information). The Li|MoS_2_@NSPCB cell shows a much smaller charge‐transfer resistance (5.5 Ω) than the Li|MoO*_x_* @NCF cell (27.2 Ω) at 30 °C, demonstrating the enhanced charge transport at the electrode/electrolyte interface. The activation energies for Li‐ion diffusion within the different hosts were calculated based on the Arrhenius plots (Figure 2e).^[^ ^26^ ^]^ The Li|MoS_2_@NSPCB cell has a much smaller activation energy of 10.89 kJ mol^−1^ than the Li|MoO_x_@NCF cell (40.80 kJ mol^−1^), suggesting that the continuous structure of the hierarchical porous nanobelts in the MoS_2_@NSPCB host allows faster Li‐ion diffusion. By virtue of the high porosity and controllable thickness, the MoS_2_@NSPCB host enables the Li|Li symmetric cells to show good performance, even at a plating/stripping capacity of 3 mAh cm^−2^ (Figure 2f). Long‐term stabilities of over 1500 and 500 h were obtained at 3 and 10 mA cm^−2^, respectively. The half‐cell performance of the Li|Li symmetric cells using the MoS_2_@NSPCB host is superior to similar cells using other hosts reported in the recent literature (Figure 2g; and Table S2, Supporting Information).^[^ ^27^, ^28^, ^29^, ^30^, ^31^, ^32^, ^33^, ^34^, ^35^ ^]^

Full cells were assembled using LiFePO_4_ (LFP) as the cathode in order to evaluate the practical performance of the MoS_2_@NSPCB host‐modified LMA (MoS_2_@NSPCB‐Li). The N/P ratio and electrolyte amount were fixed at 1:1 and ≈3.7 µL mg^−1^, respectively. The galvanostatic charge–discharge (GCD) profiles (Figure S11, Supporting Information) indicate that MoS_2_@NSPCB‐Li||LFP exhibits a smaller voltage polarization than Cu‐Li||LFP (≈63.0 mV vs ≈90.0 mV at 1 C), highlighting the faster kinetics of the MoS_2_@NSPCB host. A high reversible capacity of ≈80 mAh g^−1^ is maintained even at 10 C, showing good rate capability. The MoS_2_@NSPCB‐Li||LFP full cell can deliver a high energy of ≈460 Wh kg^−1^ based on the total weight of LFP and MoS_2_@NSPCB‐Li. Even after considering the weight of the current collectors, the MoS_2_@NSPCB‐Li||LFP full cell can deliver an energy density of 348 Wh kg^−1^, much higher than that of the Cu‐Li||LFP full cell (246 Wh kg^−1^). The MoS_2_@NSPCB‐Li||LFP pouch cell can easily power 68 LEDs and showed good bending performance by virtue of the self‐standing properties of the MoS_2_@NSPCB host (Figure 2h). Moreover, the MoS_2_@NSPCB‐Li||LFP full cell shows a remarkable cycling performance with a capacity retention of ≈70% even after 1600 cycles at 10 C (Figure 2i).

**Figure 3:**
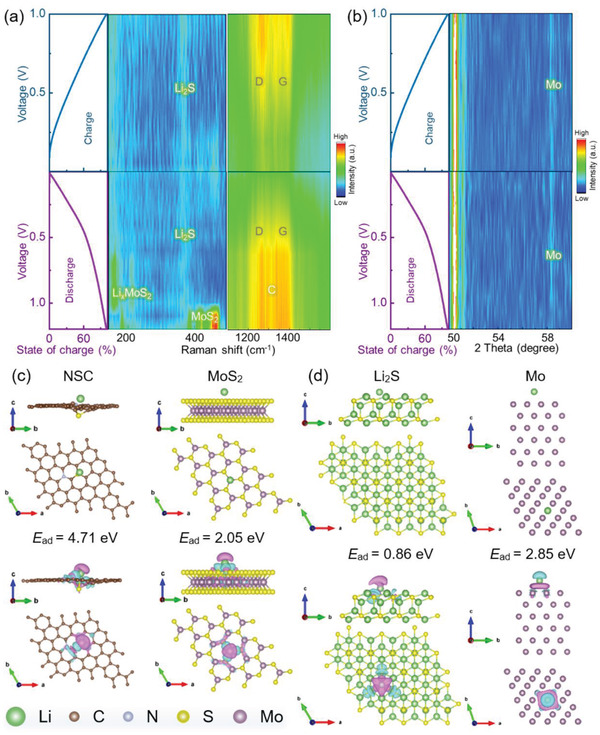
a) Galvanostatic charge–discharge (GCD) plots of the MoS_2_@NSPCB host at 0.1 A g^−1^ and the corresponding in situ Raman spectra. b) GCD plots of the MoS_2_@NSPCB host at 0.1 A g^−1^ and the corresponding in situ XRD patterns. Top and side views of different geometries, real‐space charge densities, and *E*_ad_ values of Li atoms on c) NSC, MoS_2_, d) Li_2_S, Mo. The purple and blue colors, respectively, represent decreases and increases in the charge densities.

The mechanism responsible for the modification of the MoS_2_@NSPCB host in LMAs was investigated using in situ Raman and XRD measurements on half cells. As shown in **Figure** **3**a,b, MoS_2_ is gradually lithiated, initially giving Li*_x_* MoS_2_, which is finally transformed into Li_2_S and Mo during the discharge process (MoS_2_ + 4Li^+^ + 4e^−^→ Mo + 2Li_2_S). The characteristic peaks of Li_2_S and Mo are observed at low potential during the discharge process and the subsequent charge process, confirming their good stability as the active sites for regulating Li plating/stripping (Figure S14, Supporting Information). In addition, the high degree of recovery of the D and G bands in the Raman spectra suggests good reversibility of the change in the N,S‐doped carbon framework. TEM images and the corresponding selected area electron diffraction results of the cycled MoS_2_@NSPCB host further verify that the MoS_2_ derivatives are uniformly anchored on the nanobelts with good structural integrity.

DFT calculations were used to probe the lithiophilicity and stability of the active sites within the MoS_2_ @NSPCB host. Models of Li atoms adsorbed on N,S‐doped carbon (NSC), MoS_2_, Li_2_S, and Mo were constructed, and the optimized results are displayed in Figure 3c,d. The calculated adsorption energies (*E*_ad_) of NSC and MoS_2_ are 4.71 and 2.05 eV, respectively, indicating that the N/S heteroatoms and MoS_2_ are good lithiophilic sites that can adsorb Li ions on the interface between the electrode and electrolyte in the initial cycle. As compared with N/S heteroatoms, Li_2_S and Mo show a lower *E*_ad_ of 0.86 and 2.85 eV, respectively. The moderate Li adsorption of these materials is beneficial to both Li plating and stripping because of the low nucleation/diffusion barriers of Li and good chemical stability to Li.^[^ ^36^ ^]^ Although the N,S doping is not favorable for Li diffusion and striping, it enables good carbon support which firmly anchors the ideal lithiophilic sites (MoS_2_ derivatives). The structural stability of the lithiophilic sites was further revealed by comparing the *E*_ad_ of active sites on a pure carbon support (C) and NSC (Figure S15, Supporting Information). The N,S‐doped carbon has a higher *E*_ad_ than the undoped carbon, confirming that the lithiophilic sites in the MoS_2_@NSPCB host have both high surface activity and good structural stability, thus efficiently guiding uniform Li nucleation and facilitating highly reversible Li plating/stripping.^[^ ^37^ ^]^

**Figure 4:**
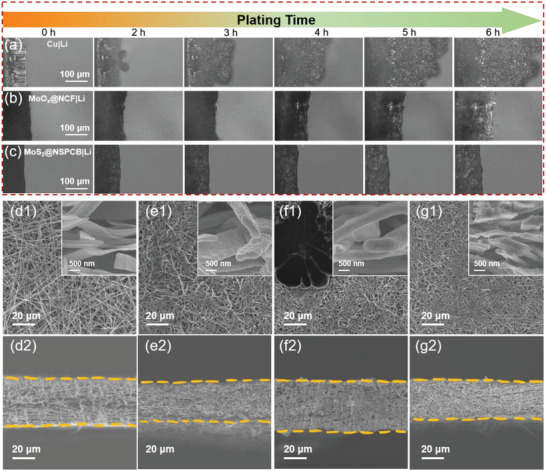
In situ optical microscopy of Li plating behavior on a) Cu foil, b) MoO_x_@NCF, and (c) MoS_2_@NSPCB at 1 mA cm^–2^ for 6 h. Ex situ surface and cross‐section SEM images of the MoS_2_@NSPCB hosts: (d1, d2) before Li plating, (e1, e2) after Li plating with a capacity of 3 mAh cm^–2^ at 1 mA cm^–2^, (f1, f2) after Li plating with a capacity of 6 mAh cm^–2^ at 1 mA cm^–2^, and (g1, g2) after Li stripping with a capacity of 6 mAh cm^–2^ at 1 mA cm^–2^.

In situ optical microscopy was further performed to investigate the inhibitory effects of the hosts on Li dendrites. As shown in **Figure** **4**a, Li dendrites formed quickly on the Cu surface, resulting in a highly porous structure with a large volume change. Although Li dendrites were not observed on the MoO*_x_* @NCF host in the initial plating process, obvious volumetric expansion and surface Li dendrite formation were eventually observed due to the limited space for Li growth (Figure 4b). In contrast, a smooth surface without any observable dendrites was observed for the MoS_2_@NSPCB host throughout the entire deposition process (Figure 4c). This shows that the self‐standing MoS_2_@NSPCB host with its abundant lithiophilic sites and 3D porous structure favors homogeneous Li plating and suppresses the formation of Li dendrites.

Ex situ SEM images for different Li plating/stripping states clearly show the morphological evolution of the MoS_2_@NSPCB host. As shown in Figure 4d, the host has an initial thickness of ≈40 µm with an ultrathin nanobelt structure; After a deposition capacity of 3 mAh cm^−2^, the host thickness of MoS_2_@NSPCB decreases due to the pressure within the cells, while the compactness of the host increases significantly (Figure 4e, 2). In addition, the thickness of the nanobelt increases from ≈100 to ≈300 nm, showing the uniform Li plating (Figure 4e, 1, inset). At a higher deposition capacity of 6 mAh cm^−2^, the host thickness increases slightly, and some Li deposits with an island‐like structure are observed on the host surface (Figure 4f). The nanobelt still exhibits a smooth surface with a thickness of 500–600 nm (Figure 4f, inset). After Li stripping with a capacity of 6 mAh cm^−2^, the thickness of the host and nanobelt decreases to ≈30 and ≈100 nm, respectively (Figure 4g). The good structural stability and high Li‐plating/stripping reversibility of the MoS_2_@NSPCB host endow the LMAs with excellent cycling performance.

## 3 Conclusions

We have fabricated a self‐standing MoS_2_@NSPCB host for LMAs using an efficient sulfate‐assisted method. LMAs using the MoS_2_@NSPCB host remain dendrite‐free with good half‐cell and full‐cell performance. A detailed physicochemical study has shown that the N,S‐doped carbon nanobelts firmly anchor the MoS_2_‐derived lithiophilic sites, which homogenize the Li nucleation and suppress the formation of Li dendrites. In addition, the presence of a conductive 3D structure with hierarchical porosity accelerates Li‐ion diffusion and accommodates the volume changes of the Li deposits. This work has not only proposed a new self‐standing host for LMAs but also suggested new ideas for the modification of other metallic anodes.

## 4 Experimental Section

### Synthesis of MoS_2_@NSPCB

An aqueous gelatin solution (15 wt%, 9 mL) was mixed with Na_2_SO_4_ (570 mg) and (NH_4_)_6_Mo_7_O_24_·4H_2_O (700 mg) by stirring at 60 °C for 1 h. The solution was used to electrospin films (ET2535X, Ucalery). The gelatin films obtained after electrospinning for 6 h were stabilized by heating at 270 °C for 1 h in air, followed by pyrolysis at 700 °C for 1 h under an argon atmosphere (heating rate: 2.5 °C min^−1^). The pyrolyzed samples were washed by water and dried overnight at 60 °C.

### Synthesis of NSPCB and MoO*_x_* @NCF

The preparation procedures were similar to that for MoS_2_@NSPCB. NSPCB and MoO*_x_* @NCF were prepared without adding (NH_4_)_6_Mo_7_O_24_·4H_2_O and Na_2_SO_4_, respectively.

## Conflict of Interest

The authors declare no conflict of interest.

## Supporting information

Supporting Information

## Data Availability

The data that support the findings of this study are available from the corresponding author upon reasonable request.
