# Supplementary material for: N,S‐Doped Porous Carbon Nanobelts Embedded with MoS2 Nanosheets as a Self‐Standing Host for Dendrite‐Free Li Metal Anodes
Source: Adv Sci (Weinh). 2022 Sep 25;9(32):2204232. doi: 10.1002/advs.202204232 (PMC9661841; doi:10.1002/advs.202204232)
Supplement: Supplementary file 1 — Supporting Information [file ADVS-9-2204232-s001.pdf]

Supporting Information

N, S-doped porous carbon nanobelts embedded with MoS_2 nanosheets as a self-standing host for dendrite-free Li metal anodes

Binke Li,[a,b,#] Weishan Cao,[a,b,#] Shuaize Wang,[a,b] Zhenjiang Cao,[a] Yongzheng Shi,[a,b] Jin Niu,[a,b,*] Feng Wang[a,b,*]

[a]State Key Laboratory of Chemical Resource Engineering, Laboratory of Electrochemical Process and Technology for Materials, Beijing University of Chemical Technology, Beijing, 100029, P. R. China

[b]Beijing Advanced Innovation Center for Soft Matter Science and Engineering, Beijing University of Chemical Technology, Beijing, 100029, P. R. China

[#]These authors contributed equally.

*Corresponding authors.

E-mail: niujin@mail.buct.edu.cn; wangf@mail.buct.edu.cn

Material Characterization

The morphology and chemical composition of the samples were investigated by SEM (JEOL, FE-JSM-6701F) and field-emission TEM (FEI, FEI Tecnai G2F30) with EDX. The crystal phase was analyzed by XRD on an Ultima IV X-Ray Diffractometer with Ni filtered Cu Kα radiation (λ = 1.5406 Å) at a voltage of 40 kV and a current of 40 mA. The surface chemical states of the samples were examined by a ESCALAB 250 XPS system with an Al Kα radiation source. Raman spectrum was collected through a LabRam HR800 using the laser excitation source at 633 nm. Thermogravimetric analysis (TGA) was carried out under air with a ramping rate of 10 °C min^{-1}. The nitrogen sorption measurement was carried on Autosorb-1 at liquid-nitrogen temperature.

Electrochemical measurements

To evaluate the half-cell and full-cell performance, CR2032 coin cell and pouch cell were assembled in an Ar-filled glovebox, respectively. Celgard 2400 membrane was used as the separator. The liquid electrolyte was composed by 1 M bis(trifluoromethanesulfonyl)imide lithium salt in 1,3-dioxolane (DOL)/1,2-dimethoxyethane (DME) with a volume ratio of 1:1 with $LiNO_3$ (2.5 wt.%) as the additive. The electrolyte amount for the half cell and full cell were 100 μL and ~3.7 μL mg^{-1} (based on the weight of electrode materials), respectively. The assembled cells were tested in a LAND multichannel battery test system.

For the Coulombic efficiency test, the MoS_2@NSPCB, MoO_x@NCF and Cu disks were (diameter: 14 mm) used as the working electrodes. Li plate (diameter: 15 mm) was used as the counter electrode. The cells were discharged under a specific current density (e.g., 1 mA cm^{-2}) for a specific Li plating capacity (e.g., 1 mAh cm^{-2}), followed by charging to 1 V at the same current density. For the symmetric cell test, the MoS_2@NSPCB, MoO_x@NCF, and Cu foil went through a pre-electrodeposition process with a Li plating capacity of 3 mAh cm^{-2} or 6 mAh cm^{-2} to obtain MoS_2@NSPCB-Li, MoO_x@NCF-Li, and Cu-Li. Then, symmetric cells were assembled using the same MoS_2@NSPCB-Li, MoO_x@NCF-Li, and Cu-Li as working and counter electrodes. The symmetric cells were cycled at different current densities

for a capacity of 1 mAh cm^{-2} or 3 mAh cm^{-2}. Full cells were assemble using the $LiFePO_4$ cathode and MoS_2@NSPCB-Li or Cu-Li with a N/P ratio of ~1. The cathode material loading is ~11.5 mg cm^{-2}. The weight of the MoS_2@NSPCB host, MoS_2@NSPCB-Li, Cu foil, Cu-Li, and Al foil are ~0.50 mg cm^{-2}, ~1.18 mg cm^{-2}, ~6.47 mg cm^{-2}, ~6.97 mg cm^{-2}, and ~4.08 mg cm^{-2}, respectively. The area of cathode and anode were ~25 cm^2 weight. The full cell was cycled at a constant rate at 4~2.7 V (1 C = 170 mA g^{-1}).

In situ Raman spectroscopy and XRD tests were carried out on the XRD diffractometer and Raman spectrometer with different electrolytic cells (Beijing Science Star Technology). The MoS_2@NSPCB hosts was used as the working electrode and Li plate was using as the counter electrode. The in situ optical microscopy was conducted on an optical microscope (XJ-550) with an electrolytic cell (Beijing Science Star Technology). MoS_2@NSPCB, MoOx@NCF, and Cu foil were used as the working electrodes and Li plate was using as the counter electrode. The thickness of the host and Cu foil were ~100 μm to get a clear observation of the morphology evolution.

First-Principles Calculation Method

The calculations were performed by the Vienna ab initio Simulation Package (VASP5.4.4) software package, taking advantage of the Projected Augmented Wave (PAW) [S1]. The exchange and correlation effect is described by the Perdew-Burke-Ernzerhof (PBE) functional under the generalized gradient approximation [S2]. The kinetic-energy cutoff of the plane-wave basis was set as 500 eV. The first Brillouin zone was sampled using 2 × 2 × 1 Γ-center Monkhorst–Pack k-points. The Gaussian smearing method was employed to calculate the electron smearing with a smearing width of 0.05 eV. The convergence threshold was set as 10^{-6} eV and 0.02 eV/Å for energy and force, respectively. The weak interactions in the system were described by the DFT-D3 (BJ) method proposed by Grimme [S3]. The construction of the heterojunction model is carried out by Vaspkit software [S4], and the calculation part of the results were used for post-processing.

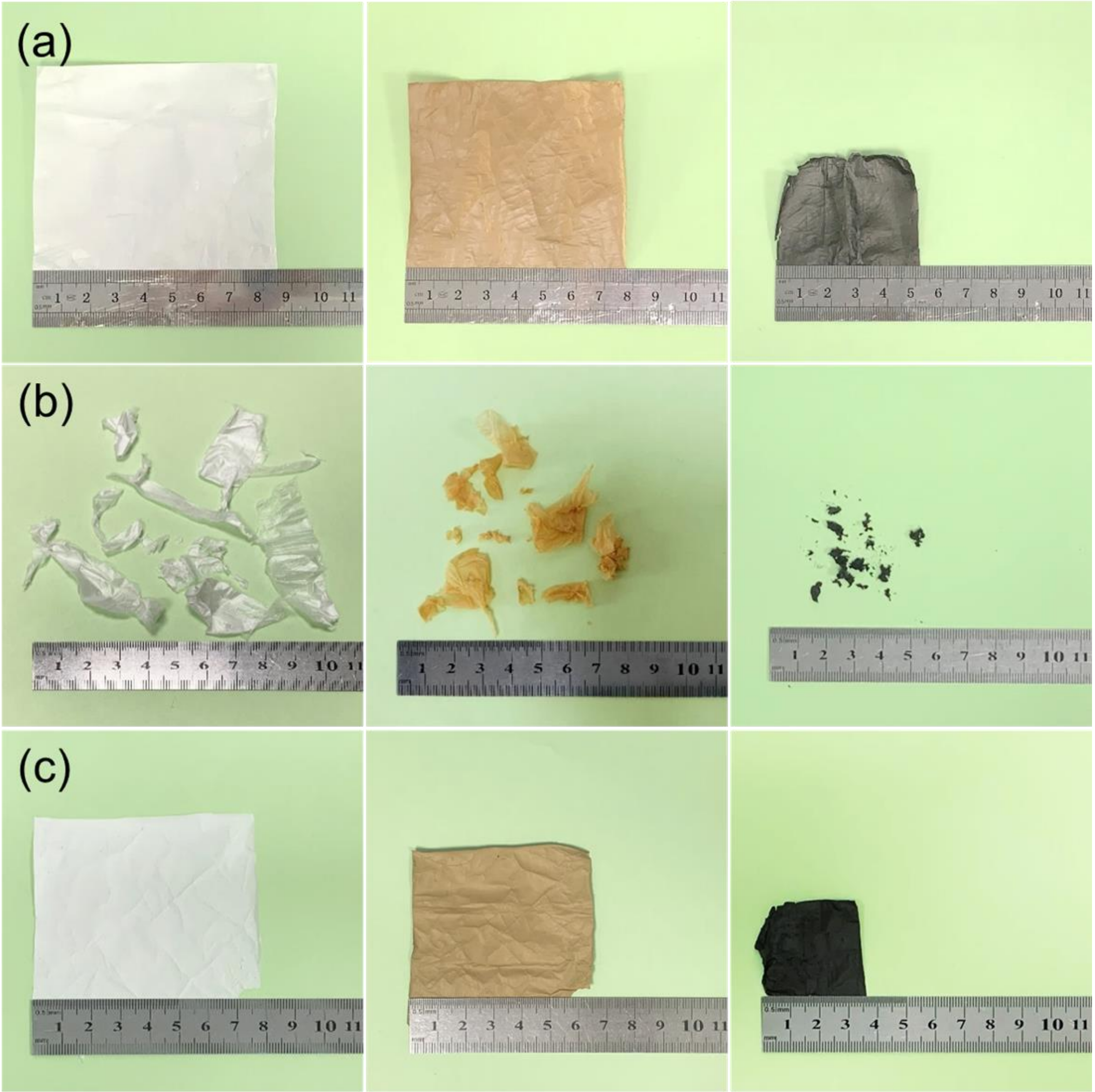

Figure S1. Digital photos of the (a) MoO_x@NCF, (b) NSPCB, and (c) MoS_2@NSPCB films obtained after electrospinning (left), pre-oxidation (middle) and carbonization (right).

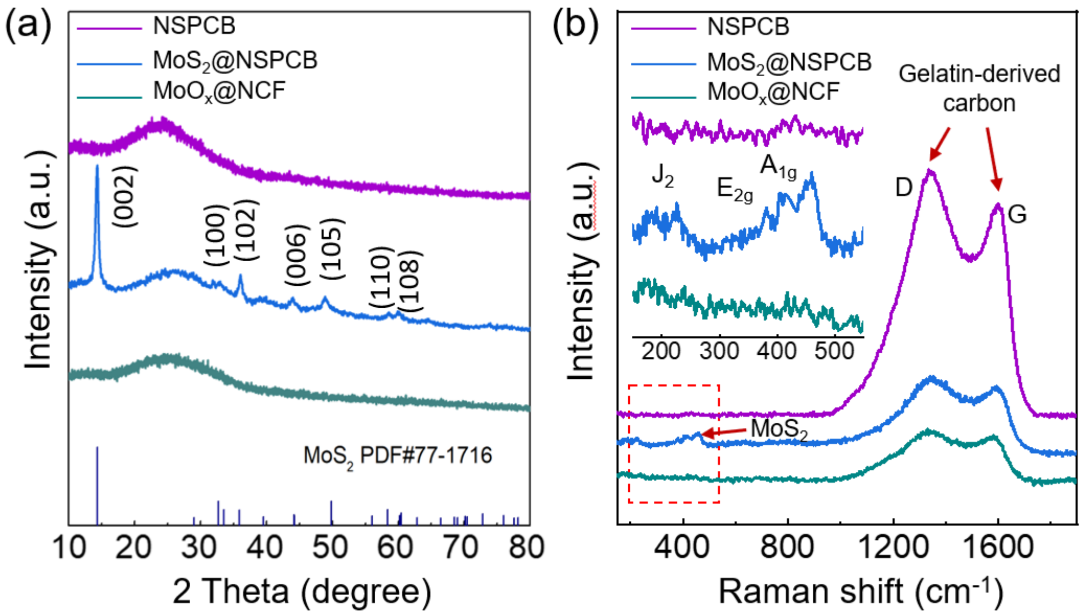

Figure S2. (a) XRD patterns and (b) Raman spectra of NSPCB, MoO_x@NCF, and MoS_2@NSPCB.

The XRD patterns and Raman spectra of NSPCB, MoO_x@NCF, and MoS_2@NSPCB are shown in Figure S2. As shown in Figure S2a, the broad and weak peaks centered at ~26º are attributed to the characteristic reflection of graphitic (002) plane, confirming the existence of gelatin-derived carbon in all the samples. In addition, obvious characteristic peaks of MoS_2 are observed in MoS_2@NSPCB, confirming the formation of MoS_2 derived from $NaSO_4$ and $(NH_4)_6Mo_7O_{24}\cdot 4H_2O$. The existence of the MoS_2 and gelatin-derived carbons are also verified by the Raman spectra which exhibit obvious characteristic bands of MoS_2 and G/D bands of carbon, respectively. No obvious peaks are observed in the XRD pattern and Raman spectrum of MoO_x@NCF, indicating the amorphous structure of the Mo compounds in MoO_x@NCF.

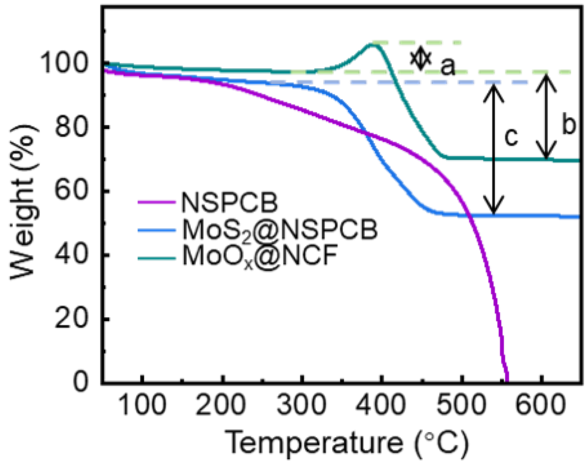

Figure S3. TG curves of NSPCB, MoO_x@NCF, and MoS_2@NSPCB in air atmosphere.

The weight content of carbons in NSPCB, MoO_x@NCF, and MoS_2@NSPCB were calculated to be ~100%, ~39.0%, and ~43.0% based on the thermogravimetric analysis in air (Figure S3).

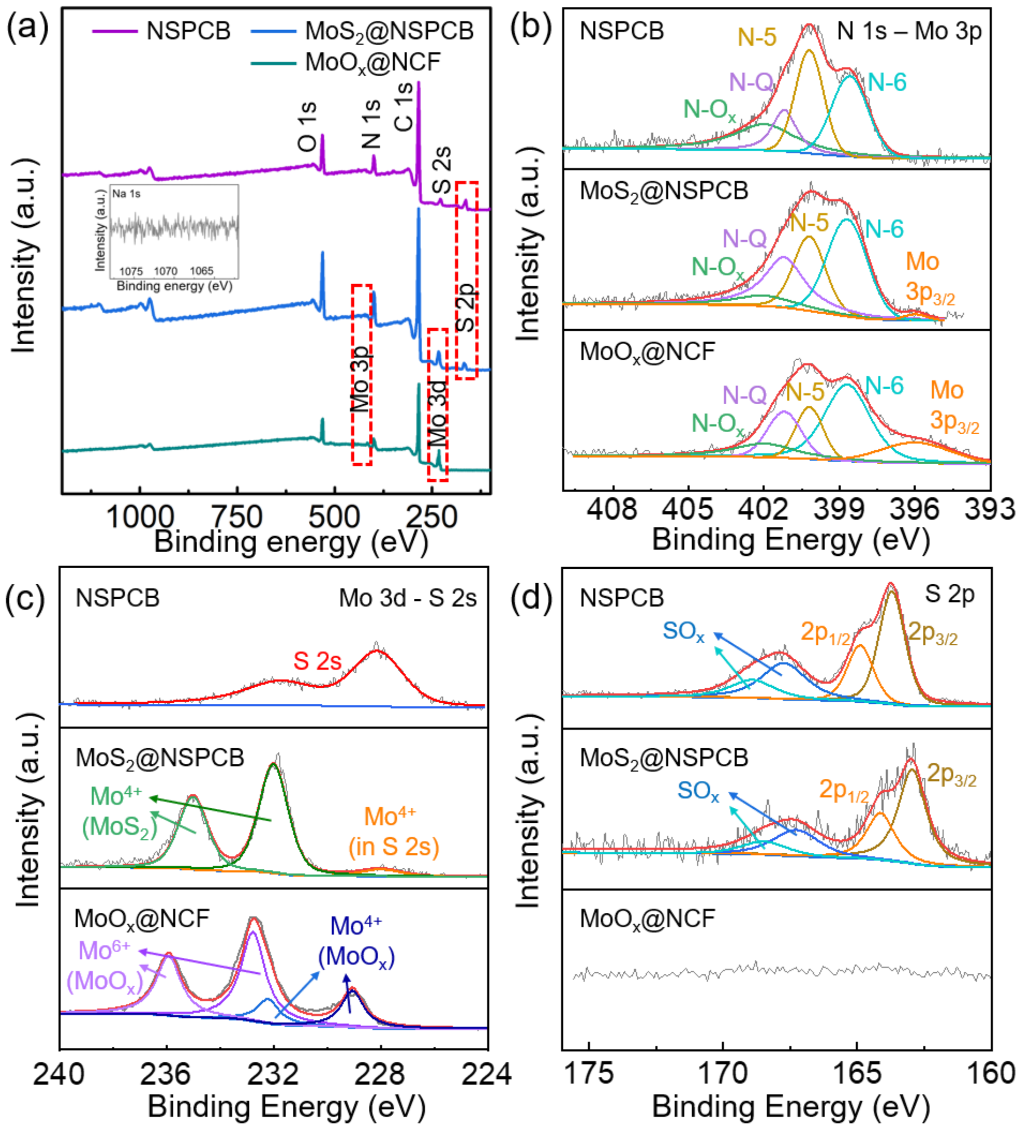

Figure S4. (a) XPS spectra (inset: high-resolution Na 1s XPS spectrum of MoS_2@NSPCB) and high-resolution (b) N 1s - Mo 3p, (c) Mo 3d - S 2s, and (d) S 2p XPS spectra of NSPCB, MoO_x@NCF, and MoS_2@NSPCB.

The elemental composition and states of three samples were analyzed by XPS. As shown in the inset of Figure S4a, there is no peak around 1072 eV which is the binding energy of Na 1s, indicating the complete removal of metallic sodium from the MoS_2@NSPCB. Obvious N species are shown in the XPS spectra of all samples, revealing the N-doping in the gelatin-derived carbons (Figure S4a, b). The N-Q, N-5, and N-6 refer to graphitic N, pyrrolic N, and pyridinic N, respectively [S5]. As shown in Figure S4c, the Mo 3d XPS spectrum of MoO_x@NCF indicates that Mo mainly

exists in the form of Mo^{6+} (MoO_x) and Mo^{4+} (MoO_x), confirming the MoO_x in MoO_x@NCF. The Mo 3d XPS spectrum of MoS_2@NSPCB indicates that Mo exists in the form of Mo^{4+} (MoS_2) of MoS_2@NSPCB, confirming the MoS_2 in MoS_2@NSPCB [S6]. In the XPS S 2p spectra (Figure S4d), the MoS2@NSPCB sample shows two typical peaks located at 162.9 and 164.1 eV, which can be attributed to the S $2p_{3/2}$ and S $2p_{1/2}$ orbitals of S^{2-} species in MoS_2, respectively [S6]. NSPCB exhibits two main peaks at 163.7 and 164.9 eV that can be assigned to the $2p_{3/2}$ and $2p_{1/2}$ of doping S atoms in carbon, respectively. This result implies that $NaSO_4$ not only acts as a sulfurizing reagent to react with molybdate to form MoS_2, but also plays a dopant role to introduce S heteroatoms into the carbon framework [S5].

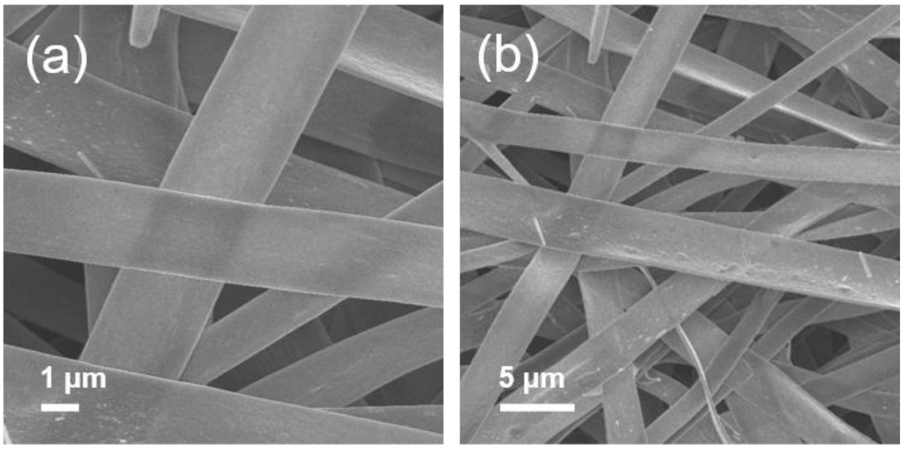

Figure S5. SEM images of MoS_2@NSPCB after electrospinning without pyrolysis.

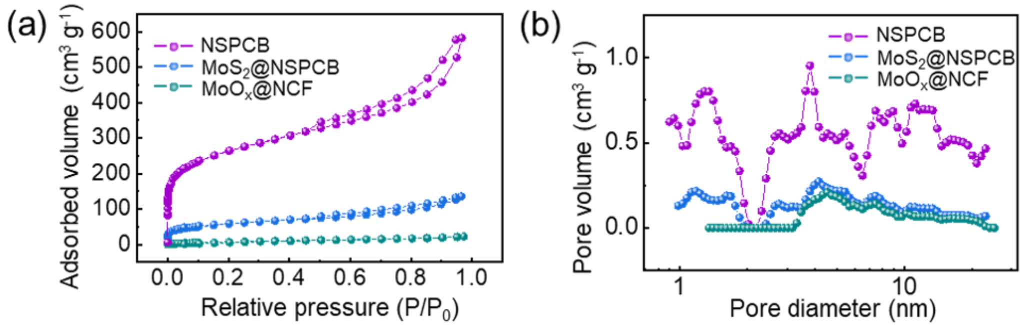

Figure S6. (a) N_2 absorption/desorption isotherm plots and (b) pore-diameter distribution plots of NSPCB, MoO_x@NCF, and MoS_2@NSPCB.

Table S1. Pore structure parameters of NSPCB, MoO_x@NCF and MoS_2@NSPCB.

			specific surface area[c] ($m^2\ g^{-1}$)	
	S_{BET}[a] ($m^2\ g^{-1}$)	V_t[b] ($cm^3\ g^{-1}$)	micropore	mesopore
NSPCB	948	0.90	448	500
MoS_2@NSPCB	210	0.21	85	125
MoO_x@NPCF	33	0.04	0	33

[a]Total specific surface area, calculated by the BET method;

[b]Total pore volume ($p/p_0 = 0.98$);

[c]Specific surface area of micropores and mesopores/macropores, calculated by the t-plot method.

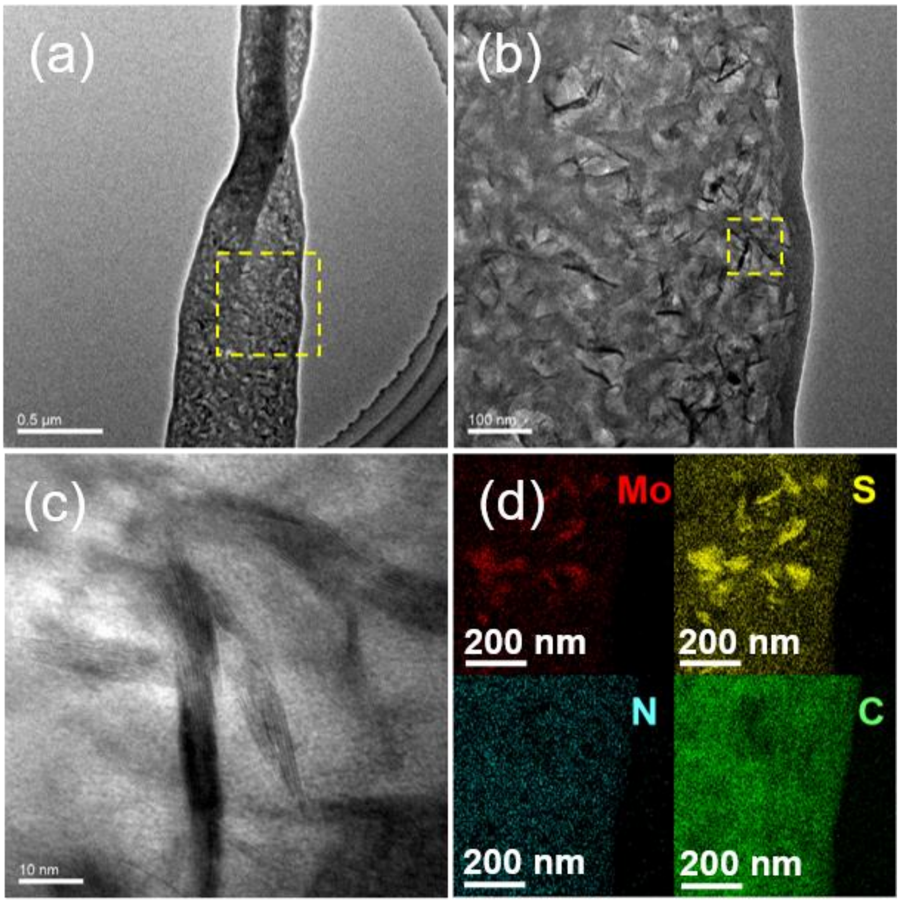

Figure S7. (a-c) TEM images of MoS_2@NSPCB at different magnifications. (d) EDX elemental mapping images of MoS_2@NSPCB.

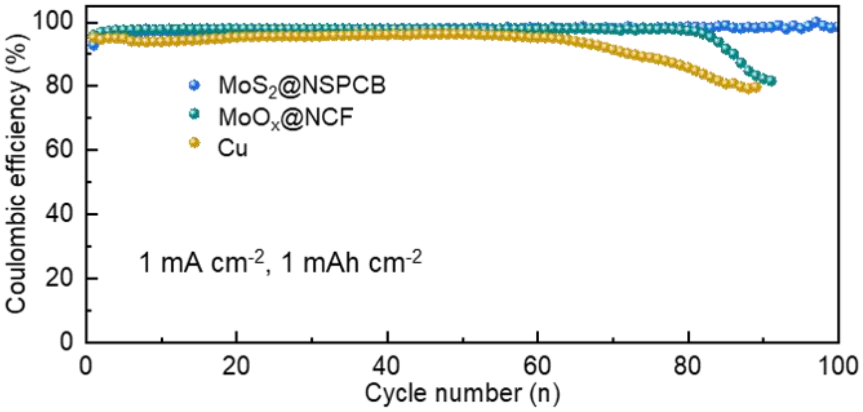

Figure S8. Coulombic efficiency of Li|Cu, Li|MoO_x@NCF, and Li|MoS_2@NSPCB half cells.

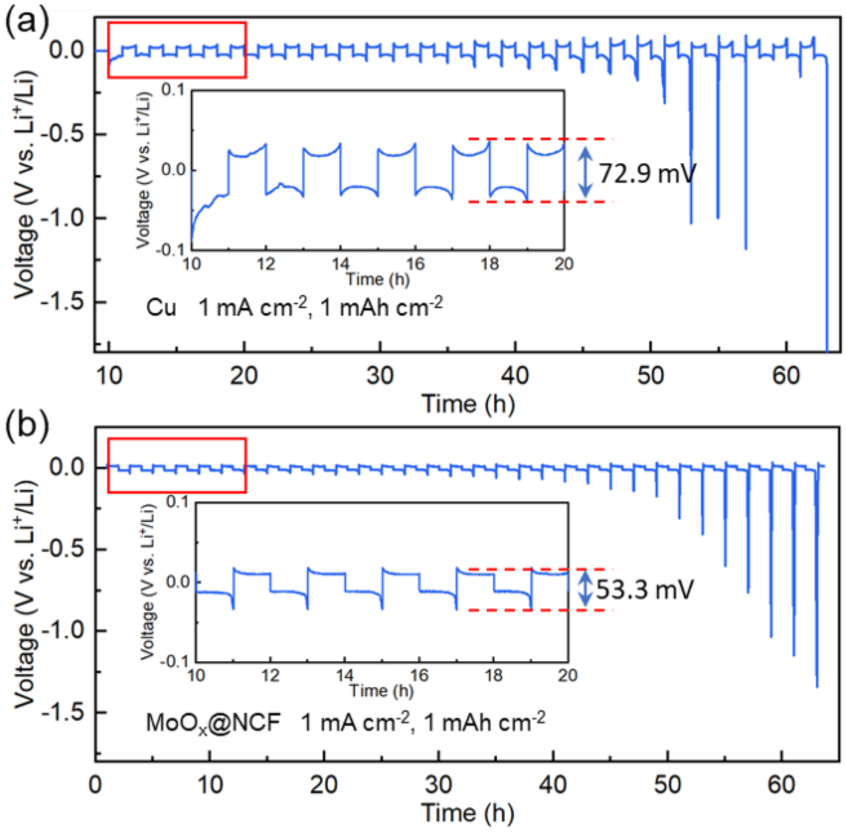

Figure S9. Galvanostatic cycling performance of the Li|Li symmetric cells using the (a) Cu and (b) MoO_x@NCF hosts.

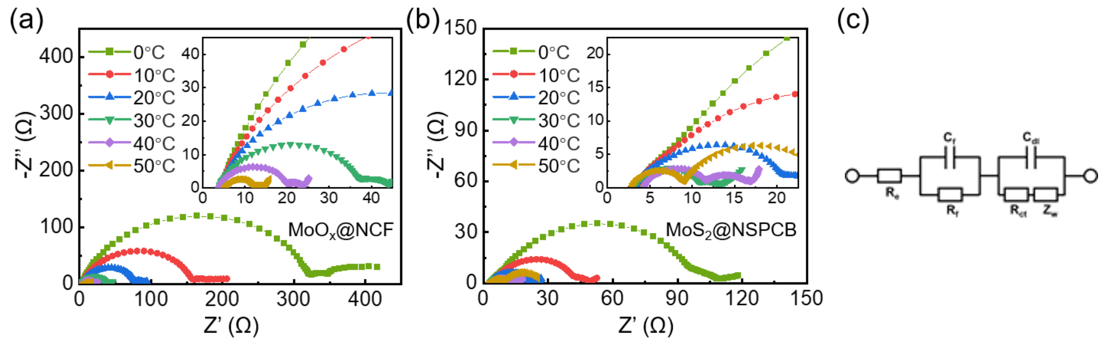

Figure S10. EIS plots at different temperatures of the (a) Li|MoO_x@NCF and (b) Li|MoS_2@NSPCB half cells. The inset is the enlarged plots at high frequency. (c) Equivalent-circuit diagram.

Table S2. Electrochemical performance comparison of the Li|Li symmetric cells using the MoS_2@NSPCB host with the cells using other hosts.

host	Current (mA cm^{-2})	Capacity (mAh cm^{-2})	Cycle time (h)	Voltage hysteresis (mV) at last cycle	Ref.[a]
**MoS_2@NSPCB**	**1**	**1**	**2000**	**~22.7**	**This work**
	**3**	**3**	**1500**	**~24.2**	
	**10**	**3**	**500**	**~41.0**	
HPSC-Li	0.5	0.5	2000	60	[27]
PDDA-TFSI@Li	1	1	1000	46	[28]
Cu@MC@Li	1	1	400	23	[29]
MLF	0.5	0.5	350	~30	[30]
Li-Mg10	0.5	1	550	~30	[31]
CC-Zn-CMFs-Li	1	1	2000	~30	[32]
h-Ti_3C_2/CNTs/Na	3	3	1200	< 80	[33]
GGCNT-Li	2	2	200	68	[34]
Li@N-TNO@Cu foil	3	1	1000	~60	[35]

[a]The reference numbers are corresponding to those in the manuscript.

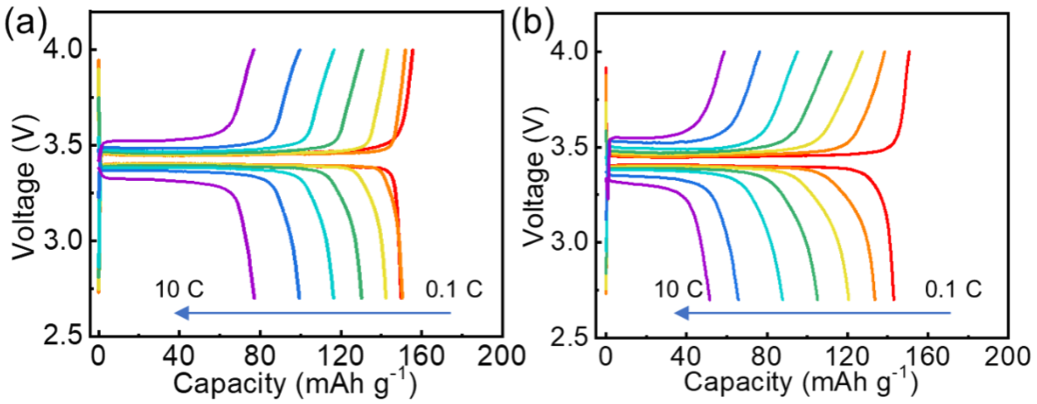

Figure S11. GCD profiles of the MoS_2@NSPCB-Li |$LiFePO_4$ and Cu-Li|$LiFePO_4$ full cells at different rates.

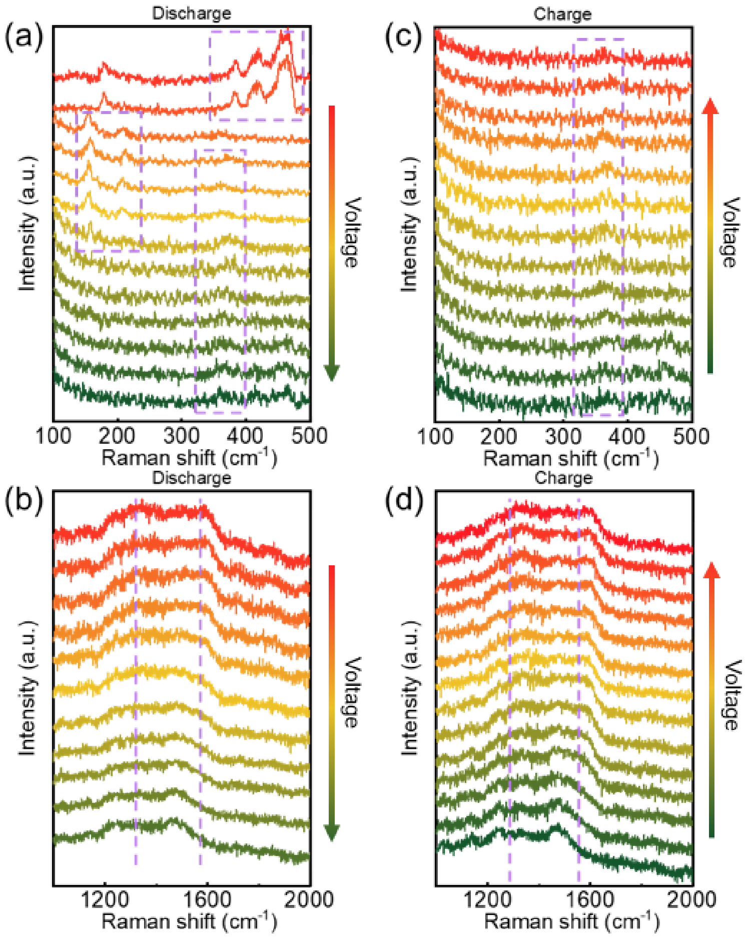

Figure S12. *In situ* Raman spectra of the MoS_2@NSPCB host during the charge-discharge process at 0.1 A g^{-1}.

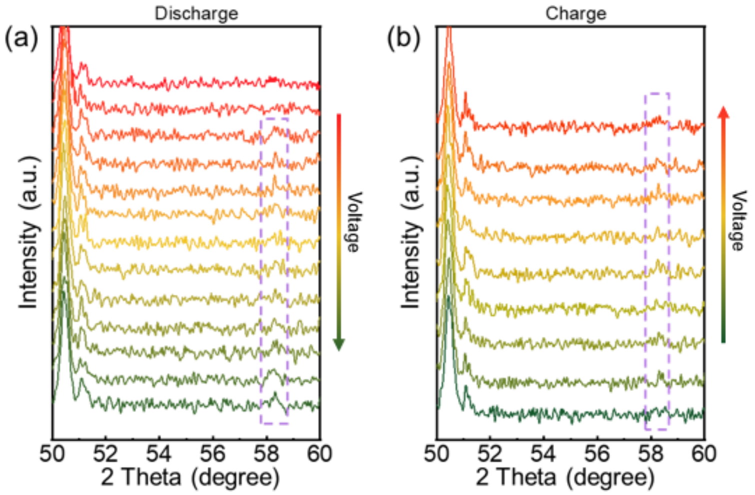

Figure S13. *In situ* XRD patterns of the MoS_2@NSPCB host during the charge-discharge process at 0.1 A g^{-1}.

The characteristic peaks of Mo are weak due to its ultrasmall crystal exhibits poor diffraction [S7].

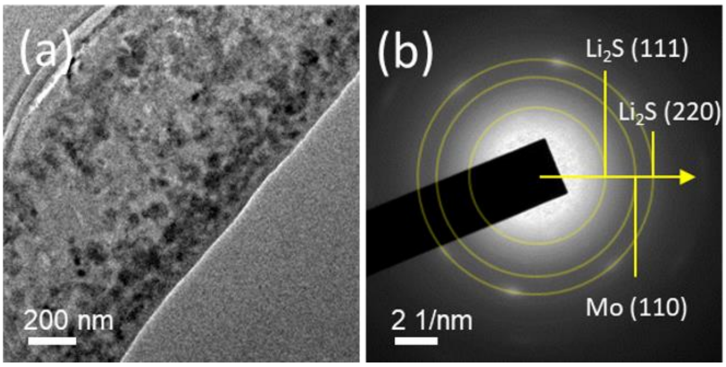

Figure S14. (a) TEM image of the MoS_2@NSPCB host after discharge and (b) the corresponding selected area electron diffraction pattern.

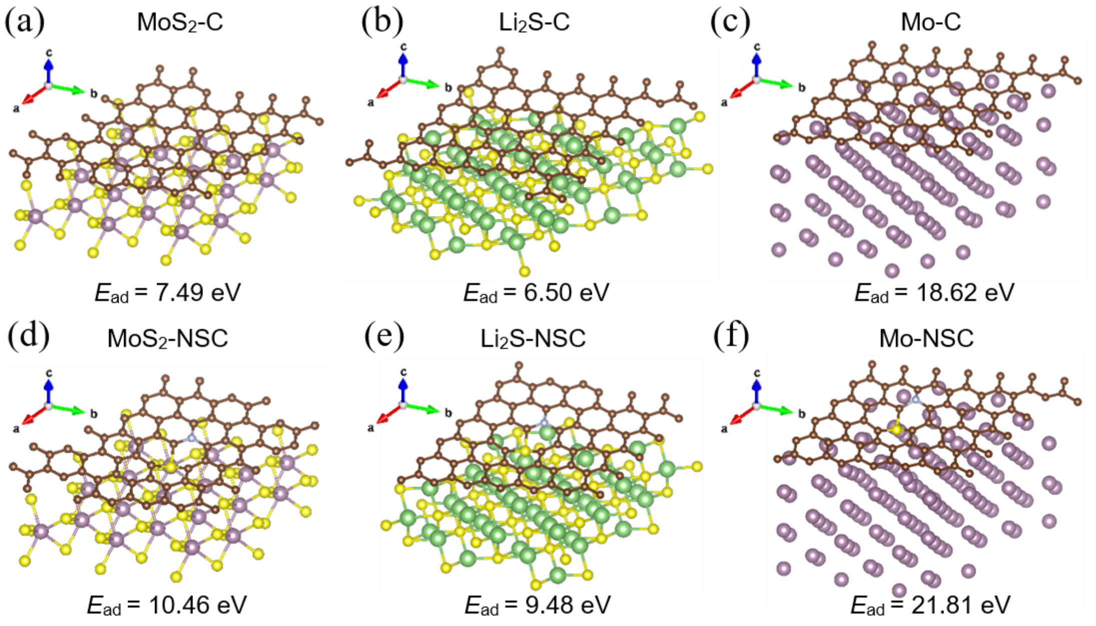

Figure S15. The side views of the geometries and E_{ad} of MoS_2, Li_2S, and Mo on (a-c) C and (d-f) NSC.

References

S1. G. Kresse, J. Furthmüller, *Phys. Rev. B* **1996**, 54 (16), 11169-11186.

S2. J. P. Perdew, K. Burke, M. Ernzerhof, *Phys. Rev. Lett.* **1996**, 77 (18), 3865-3868.

S3. S. Grimme, J. Antony, S. Ehrlich, H. Krieg, *J. Chem. Phys.* **2010**, 132 (15), 154104.

S4. V. Wang, N. Xu, J. C. Liu, G. Tang, W. T. Geng, *Comput. Phys. Commun.* **2021**, 267, 108033.

S5. M. Liu, F. Zhu, W. Cao, W. Song, J. Liu, X. Feng, Z. Li, Y. Cao, P. Wang, J. Niu, *J. Mater. Chem. A* **2022**, 10 (17), 9612-9620.

S6. S. M. Lee, J. Kim, J. Moon, K. Jung, J. H. Kim, G. J. Park, J. H. Choi, D. Y. Rhee, J. S. Kim, J. W. Lee, M. S. Park, *Nat. Commun.* **2021**, 12 (39).

S7. L. Wang, Q. Zhang, J. Zhu, X. Duan, Z. Xu, Y. Liu, H.Yang, B. Lu, *Energy Storage Mater.* **2019**, 16, 37-45.
